# Factors associated with early resumption of sexual intercourse among postnatal women in Uganda

**DOI:** 10.1186/s12978-015-0089-5

**Published:** 2015-11-19

**Authors:** Alice C. Alum, Irene B. Kizza, Charles P. Osingada, Godfrey Katende, Dan K. Kaye

**Affiliations:** Department of Nursing, School of Health Sciences, College of Health Sciences, Makerere University, P.O. Box 7072, Kampala, Uganda; Adult Health and Critical Care Department, College of Nursing, Sultan Qaboos University, P.O. Box 66, Alkhod, PC 123 Muscat, Oman; Department of Obstetrics and Gynecology, School of Medicine, College of Health Sciences, Makerere University, P.O. Box 7072, Kampala, Uganda

## Abstract

**Background:**

Despite being a key component to be addressed during postnatal period, sexuality has long been a subject of secrecy and taboo in Africa. Resumption of sexual intercourse after giving birth has been shown to reduce extramarital affairs and consequently reduce risk of sexually transmitted infections like HIV/AIDS. Consequences of early resumption of sexual intercourse include unwanted pregnancy, genital trauma and puerperal infection. The objective of the study was to assess prevalence and factors associated with early resumption of sexual intercourse among postnatal mothers attending postnatal clinic at a National referral Hospital in Uganda.

**Methodology:**

A cross-sectional study that employed an interviewer-administered questionnaire was conducted among 374 women who delivered six months prior to conducting the study. The independent variables included socio-demographic characteristics of the participant, socio-demographic characteristics of the spouse, perceived cultural norms, medical history, mode of delivery, and postpartum complications. The dependent variable was timing of resumption of sexual intercourse after childbirth (before or after six weeks postpartum). Data were analysed using SPSS version 16.0.

**Results:**

The study showed that 105 participants (21.6 %) had resumed sexual intercourse before 6 weeks after childbirth. The participants’ education level, occupation, and parity; education level of the spouse, age of baby and use of family planning were the factors associated with early resumption of sexual intercourse after child birth (before six weeks postpartum) (*p* < 0.05).

**Conclusion:**

Many women resumed sexual intercourse after six weeks. Women with high income, low parity, who ever-used contraception or had a spouse with high education level were more likely to have early resumption of sexual intercourse.

## Introduction

The postnatal period (the time just after delivery and through the first six weeks of life) is especially critical for newborns and mothers as most deaths of mothers and babies occur within this period [1–3]. This is an ideal time to deliver interventions that improve the health and survival of both the newborn and the mother [3]. Adong in her study about knowledge, perceptions and practices in pregnancy and childbirth in Uganda [4], reported that postnatal care services were poor or absent. In developed countries, virtually all women and their infants receive postpartum and postnatal care. Yet in developing countries, policies and programs have largely overlooked this critical time, hindering efforts to meet the Millennium Development Goals (MDGs) for maternal and child survival [3]. The World Health Organization (WHO) recommends that with limited resources, contact with the healthcare system postpartum would be at least during the first twenty four hours and before the end of the first week [2]. However, a review of postnatal services found that the nature and frequency of this care varies considerably [2, 5–10]. Indeed, many women who give birth are discharged within hours after child birth without any indication where they can obtain further care or support [11].

Sexuality is one of the key components to address during postnatal period; it has long been a subject of secrecy and taboo in Africa [12]. This is particularly true of sensitive issues such as postnatal sexual intercourse. Perceived sexual problems in the post-partum period need to be addressed [13, 14]. These are dyspareunia, lack of vaginal lubrication, difficulty in achieving orgasm, vaginal loosening, loss of sexual desire and bleeding or irritation after sexual intercourse [15]. Resumption of sexual intercourse after giving birth has been shown to allow the men to “stay more at home” (not to get extramarital affairs) which reduces the risk of sexually transmitted infections like HIV/AIDS infections [16]. The consequences of early resumption of sexual intercourse on the other hand, include unwanted pregnancy which may be undesirable especially if no contraceptive method is used after six months postpartum [16]. Moreover, postpartum abstinence is perceived as being against natural human sexuality and results in practices that could endanger family relationships and health, especially in the era of HIV/ AIDS [8, 17].

African women tend to follow established community norms and traditions in making decisions that influence resumption of sexual intercourse after giving birth [18]. Studies have shown that early initiation of sexual intercourse was observed in women who had spontaneous vaginal delivery and in those who stayed at home with their mothers, than in-laws and aunts [15]. Standard guidelines published by Ministry of Health in Uganda recommend that, mothers abstain from sexual intercourse for at least 6 weeks after child birth as the risk of infecting the baby through breast milk is higher if the mother is newly HIV positive [19]. For example, the risk of mother-to-child HIV transmission was 2.9-fold higher during the postpartum period among those who had recently acquired HIV than among those with chronic HIV infection, and 2.3-fold higher during the pregnancy/postpartum periods combined [20]. Postpartum women may be at greater risk for infections due to vaginal lesions and abrasions following labour and delivery process [21]. Anecdotal reports indicate that some mothers request not to be discharged quickly from hospital following childbirth for fear of being forced to have sexual intercourse by their partners before they heal. Additionally it has been observed that some women return to hospital with gaping episiotomies in the postnatal period, one indication that they had engaged in sexual intercourse before the perineum was completely healed [19]. Women need adequate counseling to be prepared for resumption of sexual intercourse postpartum to avoid complications such as puerperal infection and unwanted pregnancies. The extent to which postnatal mothers adhere to recommended guidelines is not documented. The purpose of our study was to assess the timing of resumption of sexual intercourse after childbirth and factors associated with early resumption. Information generated could guide service providers on how to improve the information given to women and their partners during antenatal care and after childbirth regarding postpartum resumption of sexual intercourse.

## Methods

### Study setting and participants

This cross-sectional study was conducted in the postnatal and immunisation clinics in Mulago National Referral and Teaching Hospital, Uganda. The Hospital has three postnatal and immunisation clinics, one for private clients (in lower Mulago) and the other two for general clients (in upper Mulago). About 20 mothers in the extended postpartum period (up to six months postpartum) access services every day, and the clinics run 5 days a week. The services offered include postnatal examination, health education on family planning, family planning, screening for cervical cancer, and infant immunisation and growth monitoring. The study participants were postpartum women who came for postnatal care (after six weeks postpartum) or brought their babies for immunization (within six months postpartum).

### Sample size and variables

Using an expected proportion of women who resumed sexual intercourse within the six weeks postpartum of 58 % [8], an acceptable error of 5 %, a power of 80 % and a significance level of 95 %, we estimated a sample size of 374 participants would be adequate for the study.

The study instruments included a pre-tested interviewer-administered questionnaire consisting of closed and open-ended questions. On the questionnaire, the independent variables included socio-demographic characteristics of the participant, socio-demographic characteristics of the spouse, perceived cultural norms, medical history, mode of delivery, and postpartum complications. The dependent variable was timing of resumption of sexual intercourse after childbirth (before or after six weeks postpartum). Resumption of sexual intercourse was defined as having the first penetrative sexual intercourse after childbirth. Resumption of sexual intercourse before six weeks postpartum was regarded as early resumption.

### Data analysis

Descriptive statistics were used summarise findings: means and standard deviations for numerical variables, and frequencies and percentages for categorical variables. The prevalence of early resumption of sexual intercourse was computed as the proportion of participants who resumed sexual intercourse before end of six weeks postpartum. The timing of sexual intercourse resumption was categorized into resumption before 6 weeks (early) and after 6 weeks (recommended time). To assess factors associated with early resumption of sexual intercourse, we used the student t-test for numerical variables and chi-square test for categorical variables. Binary logistic regression analysis was performed to determine the predictors of early resumption of sexual intercourse after child birth. For all analysis statistical significance was set at ≤ 0.05.

### Ethical considerations

Ethical clearance was sought from the Institutional Review Board of the School of Health Sciences Makerere University and Mulago Hospital Research and Ethics committee. Written informed consent was obtained from prospective participants before their participation. The participants were assured that their participation was totally voluntary and that if they chose not to participate in the study, it would not in any way affect the care they would receive.

## Results

Table 1 shows the socio-demographic characteristics of the participants. The majority of the mothers (88 %) were aged between 21 and 35 years, with average age of 27 years. Of the participants, 230 (61.5 %) belonged to the Baganda tribe; 211 (56.5 %) were of the Catholic religious denomination; 255(68.2 %) had attained secondary level of education; and 344 (92 %) were married or lived with spouses. One hundred and eighty four (49.3 %) had delivered their second child; and 243 (65 %) of their spouses had attained secondary level of education.

**Table 1:** Demographic characteristics (individual factors)

Item	Response	Frequency	Per cent
Age of mother (years)	15–20	39	10.4
	21–35	329	88
	36–45	6	1.6
Tribe	Ganda	230	61.5
	Banyankole	41	11
	Basoga	12	3.2
	Others	91	24.3
Religion	Protestant	97	25.9
	Roman catholic	211	56.4
	Muslim	26	7
	Others	40	10.7
Education of respondent	No formal education	11	2.9
	Primary	81	21.7
	Secondary	255	68.2
	Diploma	5	1.3
	Degree	22	5.9
Marital status	Unmarried	30	8.0
	Married/ living together	344	92.0
Occupation of respondent	House wife	209	55.9
	Self employed	117	31.3
	Civil servant	8	2.1
	Private	40	10.7
Parity of participant	First	50	13.4
	Second	184	49.3
	Third	115	30.8
	Fourth or more	24	6.4
Highest level attained by spouse	No formal education	3	0.8
	Primary	74	19.8
	Secondary	243	65.0
	Diploma	21	5.6
	Degree	33	8.8
Occupation of spouse	Self employed	209	55.9
	Peasant farmer	48	12.8
	Civil servant	49	13.1
	Private	68	18.2

### Participants’ obstetrical and sexual history

As shown in Table 2, 264 participants (70.5 %) had vaginal delivery with no tears, and 289 (77.3 %) of their babies were aged between 6 and 19 weeks old, implying that this is the period that had elapsed since delivery. Of the 374 participants, 105 (28.1 %) had already resumed sexual intercourse after childbirth. The timing of resumption of sexual intercourse ranged between 3 and 24 weeks. Among those who had resumed sexual intercourse, 23 (21.9 %) resumed before 6 weeks after childbirth and 82 (78.1 %) resumed after 6 weeks.

**Table 2:** Obstetrical history/practices

Item	Response	Frequency	Percentage
Mode of delivery	Vaginal no tearing	264	70.1
	Vaginal (episiotomy/tear) with stitches	66	17.6
	Caesarean section	44	11.8
Age of baby now	Six to 11 weeks	147	39.3
	12 to 19 weeks	142	38
	20 to 24 weeks	85	22.7
Resumed sexual intercourse	Yes	105	28.1
	No	269	71.9
Timing of resumption	Before 6 weeks	61	21.6
	After 6 weeks	221	78.4
Reason for resuming sexual intercourse	I wanted sex	57	21.6
	Partner demand	207	78.4
Persons she lives with after childbirth	Husband/partner	233	87.9
	Mother in-law	8	3.0
	Auntie	16	6.0
	Mother	8	3.0
Reason for not resuming sexual intercourse	Too tired	8	13.8
	Doctor/midwife told me not to	4	6.9
	Wanted to wait until 6 weeks	30	51.7
	Not with husband	16	27.6
Using any family planning	Yes	93	26.5
	No	258	73.5
Problems noted after resuming sexual intercourse	No problem	177	75.6
	Pain/bruises during sexual	26	11.1
	intercourse		
	Bleeding	19	8.1
	Abdominal pain	12	5.1
Breastfeeding the baby	No	16	4.3
	Yes	358	95.7
Received health education/advice on when to resume sexual intercourse	Not sure whether given	89	23.8
	No advice given	245	65.5
	Advice given	40	10.7

Among the participants who had not resumed sexual intercourse, 139 (51.7 %) wished to wait until 6 weeks, 74 (27.6 %) were not staying with the husbands, 37 (13.8 %) reported still feeling too tired, and 19 (6.9 %) reported receiving advice against early resumption from the doctor or midwife as the reasons for not resuming. There were no problems experienced by 79 (75.2 %) of the participants that had resumed sexual intercourse early (before six weeks postpartum), although some of them reported pain and/ or bruises during sexual intercourse 26 (24.8 %), bleeding 19(18.1 %), and abdominal pain 12 (11.4 %). Only 93 (26.5 %) were using a family planning method.

### Predictors of early resumption of the sexual intercourse after child birth

Using the Pearson’s chi-square test (Tables 3 and 4), predictors of early resumption of sexual intercourse after child birth were participant’s religion (*p* < 0.01), level of education (*p* < 0.01), occupation (*p* < 0.01), parity (*p* < 0.01), spouse’s level of education (*p* < 0.01), age of the baby or time since delivery (*p* < 0.01), use of family planning (*p* < 0.01), breast feeding of the baby (*p* < 0.01), and mode of delivery (*p* < 0.01). On multivariate analysis, independent predictors of early resumption of sexual intercourse (Table 5) were occupation of the mother (OR = 0.17; CI 0.09-0.34; *p* = 0.000), parity (OR = 6.92; CI 2.41-19.86; *p* = 0.000), education level of spouse (OR = 0.33; CI 0.011–0.103; *p* = 0.000) and use of family planning methods (OR =17.03; CI 2.24–127.27).

**Table 3:** Factors associated with early resumption of sexual intercourse

Variable		Timing of resumption		*P* value
		Before 6 weeks (%)	After 6 weeks (%)	
Age	15–20	1 (3.3)	17 (7.7)	0.223
	21–35	54 (88.5)	203 (91.9)	
	36–45	5 (8.2)	1(1.6)	
Religion	Protestant	8 (13.1)	79 (35.7)	0.000
	Roman catholic	45 (73.8)	119 (53.8)	
	Muslim	8 (13.1)	8 (3.6)	
	Others	0 (0)	15 (6.8)	
Level of education by respondent	No formal education	2 (3.3)	6 (2.7)	0.000
	Primary	22 (36.1)	52 (23.5)	
	Secondary	21 (34.4)	161 (72.9)	
	Diploma	1 (1.6)	2 (0.9)	
	Degree	15 (24.6)	0 (0)	
Marital status	Unmarried	3 (4.9)	15 (6.8)	0.772
	Married	58 (95.1)	206 (93.2)	
Occupation of respondent	House wife	21 (34.4)	163 (73.8)	0.000
	Self employed	24 (39.3)	42 (19.0)	
	Civil servant	8 (13.1)	1 (0.5)	
	Private	8 (13.1)	15 (6.8)	
Parity	First	0 (0)	24 (10.9)	0.000
	Second	61 (100)	76 (34.4)	
	Third or more	0 (0)	121 (54.7)	
Highest level by spouse	No formal education	0 (0)	2 (0.9)	0.000
		16 (26.2)	58 (26.2)	
	Primary			
		16 (26.2)	161 (72.9)	
	Secondary			
		21 (34.4)	0 (0)	
	Diploma			
		8 (13.1)	0 (0)	
	Degree

**Table 4:** Obstetrical factors associated with early resumption of sexual intercourse

Variable		Timing of resumption		*P* value
		Before 6 weeks (%)	After 6 weeks (%)	
Mode of delivery	Vaginal no tearing	53 (86.9)	152 (68.8)	0.009
	Vaginal with (episiotomy/tear )	8 (13.1)	50 (22.6)	
	Caesarean section	0 (0.0)	19 (8.6)	
Time from childbirth	Less than 6 weeks		39 (17.4)	0.000
	6–11 weeks	45 (73.8)	182 (82.4)	
	12–24 weeks	16 (26.2)		
Using family planning	No	16 (26.2)	183 (85.5)	0.000
	Yes	45 (73.8)	31 (14.5)	
Breast feeding the baby	No	8 (13.1)	0 ( 0.0)	0.000
	Yes	53 (86.9)	221 (100.0)	
Breastfeeding the baby exclusively	No	11 (18)	36 (15.8)	0.681
	Yes	50 (82)	186 (84.2)

**Table 5:** Logistic regression for early resumption of sexual intercourse after child birth on individual factors

Characteristic	Odds Ratios	95.0 % CI	*P*-value
Education	1.69	0.67–4.23	0.265
Occupation	0.17	0.09–0.34	0.000
Parity	6.92	2.41–19.87	0.000
Level of education of spouse	0.03	0.01–0.10	0.000
Occupation of spouse	1.22	0.82–1.82	0.324
Time from childbirth	3.74	1.18–11.86	0.025
Use of family planning	17.03	2.24–127.27	0.006

## Discussion

Many women had resumed sexual intercourse by 6 weeks postpartum. The finding is similar to that of others [9, 10, 22] who reported that the earliest time for resumption of sexual intercourse was 3 weeks while the latest time was at 13 weeks postpartum. Similarly, a study conducted in Uganda among women with HIV reported that 58 % of women had resumed sexual intercourse within 6 weeks after childbirth [8]. Our study is in agreement with Perry [6], who reported that sexual intercourse can be resumed safely by the second to fourth week after birth, when bleeding has stopped and the episiotomy or laceration site has healed.

Our study shows that occupation of the mother was significantly associated with timing of resumption of sexual intercourse. This finding is similar to that of Radziah et al. [9] from Malaysia who found that mothers who earned more money had early resumption of sexual intercourse during their postpartum period compared to those who earned less money. Therefore, it can be inferred from other studies and ours that higher income is associated with early sexual resumption of sexual intercourse (before six weeks postpartum).

The finding that parity was significantly associated with timing of resumption of sexual intercourse after child birth is in agreement with others [23] who found that women with few children resume sexual intercourse earlier than women who had many children. Regarding education level of spouse, our finding of a significant association with early resumption of sexual intercourse is similar to that of Osinde et al. [8], where women with male partners with a higher educational level resumed sexual intercourse earlier than six weeks postpartum.

Likewise, as in the study by Rowland et al. [23], the age of the baby was significantly associated with early resumption of sexual intercourse after child birth is similar to that of Rowland et al. [23]. The finding that use of family planning was associated with early resumption of sexual intercourse after child birth is similar to that of other studies [8, 9, 17, 23–25]. The finding is contrary other studies [8, 26] which reported no differences in resumption of sexual intercourse among the women who were using postpartum contraception compared with those who were not.

Religion and cultural norms influence the timing of resumption of sexual intercourse, as noted in the study. Religion influenced resumption of sexual intercourse in a study from Cote d’Ivore [18] and the Gambia [18], where Islamic traditions prescribe 40 days of postpartum abstinence. A similar custom was reported in the Gambia in a predominantly Muslim population [18]. In Nigeria, 1 % of women resume sexual intercourse due to cultural demand [10]. A study from Uganda [22] reported that different tribal customs influenced the timing of resumption of sexual intercourse within the early days of puerperium. In this study, some women resumed sexual intercourse early because they were fulfilling cultural demands. For instance, in some cultures it was believed that a woman was expected to resume sexual intercourse within the first week after delivery ‘’so as to help in the healing of the wounds” and ‘’to bring good health to the baby” [22]. The cultural practice of new mothers staying with their in laws or at their parents’ homes after childbirth was also one way in which culture prohibited early resumption of sexual intercourse after childbirth [9, 10, 15, 22]. In Tanzania, women were sometimes described as ‘giving in’ to men’s sexual advances in order to protect their marriage, avoid divorce, or family violence, such as battering and rape, as men force them to have sexual intercourse against their will’ [13]. Spousal pressure and fear that their spouse would leave them were cited as reasons for early resumption of sexual intercourse [8].

The mode of delivery might influence timing of resumption of sexual intercourse. Women who had vaginal delivery with stitches were far less likely to resume intercourse early compared to women who had a vaginal delivery without perineal stitches or who had caesarean sections [15, 22, 23]. Women who had a vaginal delivery were 3.6 times more likely to experience sexual problem(s) on resumption of sexual intercourse postpartum compared with those that delivered by caesarean section [10]. A study from India [25] found that the median time to restart intercourse after a normal vaginal delivery with episiotomy was 40 days postpartum compared to 10 days postpartum and after a caesarean section. The most common problems in the normal delivery group were decreased libido (80 %), sexual dissatisfaction (65 %), and vaginal looseness (55 %), while in the caesarean section group, the most common problems were vaginal dryness (85 %), sexual dissatisfaction (60 %), and decreased libido (35 %) [24]. Occurrence of dyspareunia in the postpartum year was more likely in women who had a vaginal delivery [22–25]. In contrast, a study from Iran [7] found no association between the mode of delivery and timing of resumption of sexual intercourse or dyspareunia during 2–6 weeks postpartum. While interest in sexual activity often decreases throughout pregnancy, it eventually returns to normal postpartum with average resumption of intercourse, ranging between 5 and 8 weeks after childbirth [27–29].

## Conclusion

Over one in every five mothers resumed sexual intercourse before the recommended six weeks postpartum. Occupation of the mother, parity, level of education of the spouse, time from delivery and use of family planning were the significant determinants of early resumption of sexual intercourse after child birth.
